# Securing Machine Learning in the Cloud: A Systematic Review of Cloud Machine Learning Security

**DOI:** 10.3389/fdata.2020.587139

**Published:** 2020-11-12

**Authors:** Adnan Qayyum, Aneeqa Ijaz, Muhammad Usama, Waleed Iqbal, Junaid Qadir, Yehia Elkhatib, Ala Al-Fuqaha

**Affiliations:** ^1^ Information Technology University (ITU), Lahore, Pakistan; ^2^ AI4Networks Research Center, University of Oklahoma, Norman, OK, United States; ^3^ Social Data Science (SDS) Lab, Queen Mary University of London, London, United Kingdom; ^4^ School of Computing and Communications, Lancaster University, Lancaster, United Kingdom; ^5^ Hamad Bin Khalifa University (HBKU), Doha, Qatar

**Keywords:** Machine Learning as a Service, cloud-hosted machine learning models, machine learning security, cloud machine learning security, systematic review, attacks, defenses

## Abstract

With the advances in machine learning (ML) and deep learning (DL) techniques, and the potency of cloud computing in offering services efficiently and cost-effectively, Machine Learning as a Service (MLaaS) cloud platforms have become popular. In addition, there is increasing adoption of third-party cloud services for outsourcing training of DL models, which requires substantial costly computational resources (e.g., high-performance graphics processing units (GPUs)). Such widespread usage of cloud-hosted ML/DL services opens a wide range of attack surfaces for adversaries to exploit the ML/DL system to achieve malicious goals. In this article, we conduct a systematic evaluation of literature of cloud-hosted ML/DL models along both the important dimensions—*attacks* and *defenses*—related to their security. Our systematic review identified a total of 31 related articles out of which 19 focused on attack, six focused on defense, and six focused on both attack and defense. Our evaluation reveals that there is an increasing interest from the research community on the perspective of attacking and defending different attacks on Machine Learning as a Service platforms. In addition, we identify the limitations and pitfalls of the analyzed articles and highlight open research issues that require further investigation.

## 1 Introduction

In recent years, machine learning (ML) techniques have been successfully applied to a wide range of applications, significantly outperforming previous state-of-the-art methods in various domains: for example, image classification, face recognition, and object detection. These ML techniques—in particular deep learning (DL)–based ML techniques—are resource intensive and require a large amount of training data to accomplish a specific task with good performance. Training DL models on large-scale datasets is usually performed using high-performance graphics processing units (GPUs) and tensor processing units. However, keeping in mind the cost of GPUs/Tensor Processing Units and the fact that small businesses and individuals cannot afford such computational resources, the training of deep models is typically outsourced to clouds, which is referred to in the literature as *“Machine Learning as a Service”* (MLaaS).

**FIGURE 1:**
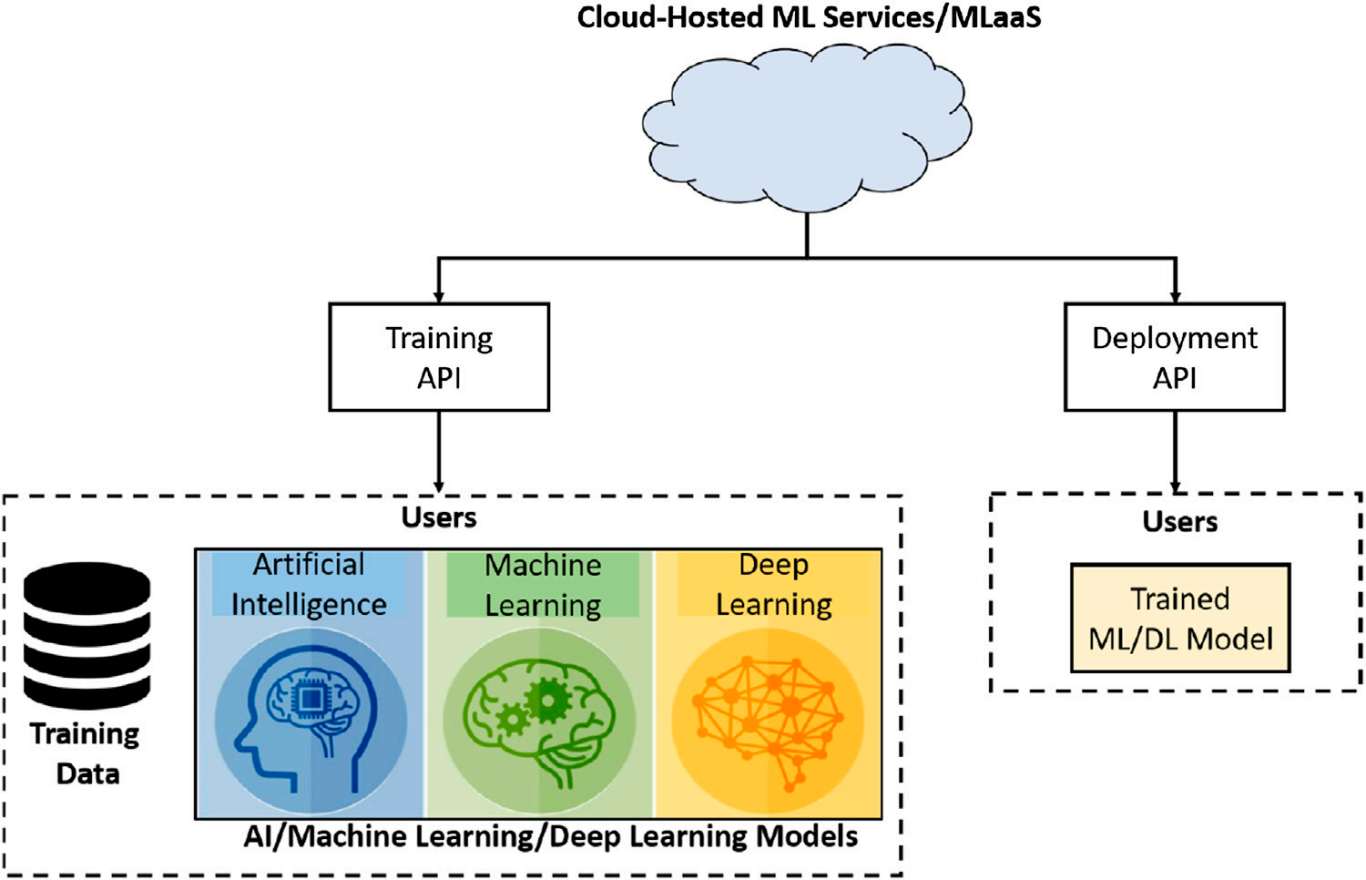
Taxonomy of different defenses proposed for defending attacks on the third-party cloud-hosted machine learning (ML) or deep learning (DL) models.

MLaaS refers to different ML services that are offered as a component of a cloud computing services, for example, predictive analytics, face recognition, natural language services, and data modeling APIs. MLaaS allows users to upload their data and model for training at the cloud. In addition to training, cloud-hosted ML services can also be used for inference purposes, that is, models can be deployed on the cloud environments; the system architecture of a typical MLaaS is shown in Figure 1.

MLaaS^1^ can help reduce the entry barrier to the use of ML and DL through access to managed services of wide hardware heterogeneity and incredible horizontal scale. MLaaS is currently provided by several major organizations such as Google, Microsoft, and Amazon. For example, Google offers Cloud ML Engine^2^ that allows developers and data scientists to upload training data and model which is trained on the cloud in the *Tensorflow* ^3^ environment. Similarly, Microsoft offers Azure Batch AI^4^—a cloud-based service for training DL models using different frameworks supported by both Linux and Windows operating systems and Amazon offers a cloud service named Deep Learning AMI (DLAMI)^5^ that provides several pre-built DL frameworks (e.g., MXNet, Caffe, Theano, and Tensorflow) that are available in Amazon’s EC2 cloud computing infrastructure. Such cloud services are popular among researchers as evidenced by the price lifting of Amazon’s p2.16x large instance to the maximum possible—two days before the deadline of NeurIPS 2017 (the largest research venue on ML)—indicating that a large number of users request to reserve instances.

In addition to MLaaS services that allow users to upload their model and data for training on the cloud, *transfer learning* is another strategy to reduce computational cost in which a pretrained model is fine-tuned for a new task (using a new dataset). Transfer learning is widely applied for image recognition tasks using a convolutional neural network (CNN). A CNN model learns and encodes features like edges and other patterns. The learned weights and convolutional filters are useful for image recognition tasks in other domains and state-of-the-art results can be obtained with a minimal amount of training even on a single GPU. Moreover, various popular pretrained models such as AlexNet (Krizhevsky et al., 2012), VGG (Simonyan and Zisserman, 2015), and Inception (Szegedy et al., 2016) are available for download and fine-tuning online. Both of the aforementioned outsourcing strategies come with new security concerns. In addition, the literature suggests that different types of attacks can be realized on different components of the communication network as well (Usama et al., 2020a), for example, intrusion detection (Han et al., 2020; Usama et al., 2020b), network traffic classification (Usama et al., 2019), and malware detection systems (Chen et al., 2018). Moreover, adversarial ML attacks have also been devised for client-side ML classifiers, that is, Google’s phishing pages filter (Liang et al., 2016).

*Contributions of the article:* In this article, we analyze the security of MLaaS and other cloud-hosted ML/DL models and provide a systematic review of associated security challenges and solutions. To the best of our knowledge, this article is the first effort on providing a systematic review of the security of cloud-hosted ML models and services. The following are the major contributions of this article:

1. We conducted a systematic evaluation of 31 articles related to MLaaS attacks and defenses.
2. We investigated five themes of approaches aiming to attack MLaaS and cloud-hosted ML services.
3. We examined five themes of defense methods for securing MLaaS and cloud-hosted ML services.
4. We identified the pitfalls and limitations of the examined articles. Finally, we have highlighted open research issues that require further investigation.

*Organization of the article:* The rest of the article is organized as follows. The methodology adopted for the systematic review is presented in Section 2. The results of the systematic review are presented in Section 3. Section 4 presents various security challenges associated with cloud-hosted ML models and potential solutions for securing cloud-hosted ML models are presented in Section 5. The pitfalls and limitations of the reviewed approaches are discussed in Section 6. We briefly reflect on our methodology to identify any threats to the validity in Section 8 and various open research issues that require further investigation are highlighted in Section 7. Finally, we conclude the article in Section 9.

## 2 Review Methodology

**FIGURE 2:**
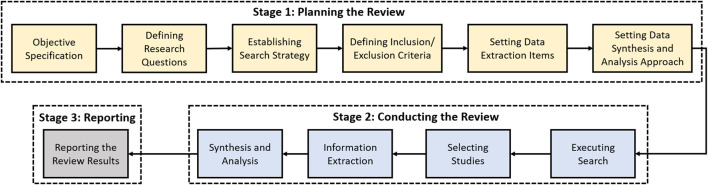
An illustration of a typical cloud-based ML or machine learning as a service (MLaaS) architecture.

In this section, we present the research objectives and the adopted methodology for the systematic review. The purpose of this article is to identify and systematically review the state-of-the art research related to the security of the cloud-based ML/DL techniques. The methodology followed for this study is depicted in Figure 2.

### 2.1 Research Objectives

The following are the key objectives of this article.

O1: To build upon the existing work around the security of cloud-based ML/DL methods and present a broad overview of the existing state-of-the-art literature related to MLaaS and cloud-hosted ML services.

O2: To identify and present a taxonomy of different attack and defense strategies for cloud-hosted ML/DL models.

O3: To identify the pitfalls and limitations of the existing approaches in terms of research challenges and opportunities.

### 2.2 Research Questions

To achieve our objectives, we consider answering two important questions that are described below and conducted a systematic analysis of 31 articles.

Q1: What are the well-known attacks on cloud-hosted/third-party ML/DL models?

Q2: What are the countermeasures and defenses against such attacks?

### 2.3 Review Protocol

We developed a review protocol to conduct the systematic review; the details are described below.

#### 2.3.1 Search Strategy and Searching Phase

To build a knowledge base and extract the relevant articles, eight major publishers and online repositories were queried that include ACM Digital Library, IEEE Xplore, ScienceDirect, international conference on machine learning, international conference on learning representations, journal of machine learning research, neural information processing systems, USENIX, and arXiv. As we added non-peer–reviewed articles from electric preprint archive (arXiv), we (AQ and AI) performed the critical appraisal using AACODS checklist; it is designed to enable evaluation and appraisal of gray literature (Tyndall, 2010), which is designed for the critical evaluation of gray literature.

**FIGURE 3:**
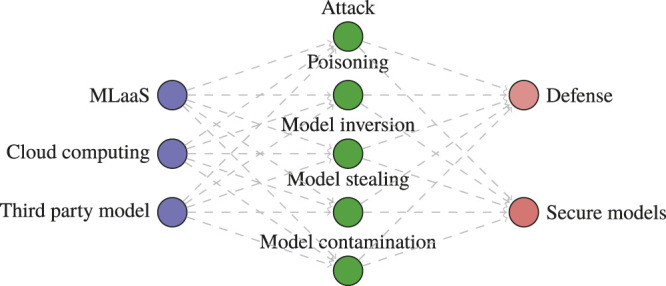
The methodology for systematic review.

In the initial phase, we queried main libraries using a set of different search terms that evolved using an iterative process to maximize the number of relevant articles. To achieve optimal sensitivity, we used a combination of words: attack, poisoning, Trojan attack, contamination, model inversion, evasion, backdoor, model stealing, black box, ML, neural networks, MLaaS, cloud computing, outsource, third party, secure, robust, and defense. The combinations of search keywords used are depicted in Figure 3. We then created search strategies with controlled or index terms given in Figure 3. Please note that no lower limit for the publication date was applied; the last search date was June 2020. The researchers (WI and AI) searched additional articles through citations and by snowballing on Google Scholar. Any disagreement was adjudicated by the third reviewer (AQ). Finally, articles focusing on the attack/defense for cloud-based ML models were retrieved.

#### 2.3.2 Inclusion and Exclusion Criteria

The inclusion and exclusion criteria followed for this systematic review are defined below.

##### 2.3.2.1 Inclusion Criteria

The following are the key points that we considered for screening retrieved articles as relevant for conducting a systematic review.

- We included all articles relevant to the research questions and published in the English language that discusses the attacks on cloud-based ML services, for example, offered by cloud computing service providers.
- We then assessed the eligibility of the relevant articles by identifying whether they discussed either attack or defense for cloud-based ML/DL models.
- Comparative studies that compare the attacks and robustness against different well-known attacks on cloud-hosted ML services (poisoning attacks, black box attacks, Trojan attacks, backdoor attacks, contamination attacks, inversion, stealing, and invasion attacks).
- Finally, we categorized the selected articles into three categories, that is, articles on attacks, articles on defenses, and articles on attacks and defenses.

##### 2.3.2.2 Exclusion Criteria

The exclusion criteria are outlined below.

- Articles that are written in a language other than English.
- Articles not available in full text.
- Secondary studies (e.g., systematic literature reviews, surveys, editorials, and abstracts or short papers) are not included.
- Articles that do not discuss attacks and defenses for cloud-based/third-party ML services, that is, we only consider those articles which have proposed an attack or defense for a cloud-hosted ML or MLaaS service.

#### 2.3.3 Screening Phase

For the screening of articles, we employ two phases based on the content of the retrieved articles: 1) title and abstract screening and 2) full text of the publication. Please note that to avoid bias and to ensure that the judgment about the relevancy of articles is entirely based on the content of the publications, we intentionally do not consider authors, publication type (e.g., conference and journal), and publisher (e.g., IEEE and ACM). Titles and abstracts might not be true reflectors of the articles’ contents; however, we concluded that our review protocol is sufficient to avoid provenance-based bias.

It is very common that the same work got published in multiple venues, for example, conference papers are usually extended to journals. In such cases, we only consider the original article. In the screening phase, every article was screened by at least two authors of this article that were tasked to annotate the articles as either relevant, not relevant, or need further investigation, which was finalized by the discussion between the authors until any such article is either marked relevant or not relevant. Only original technical articles are selected, while survey and review articles are ignored. Finally, all selected publications were thoroughly read by the authors for categorization and thematic analysis.

## 3 Review Results

### 3.1 Overview of the Search and Selection Process Outcome

**FIGURE 4:**
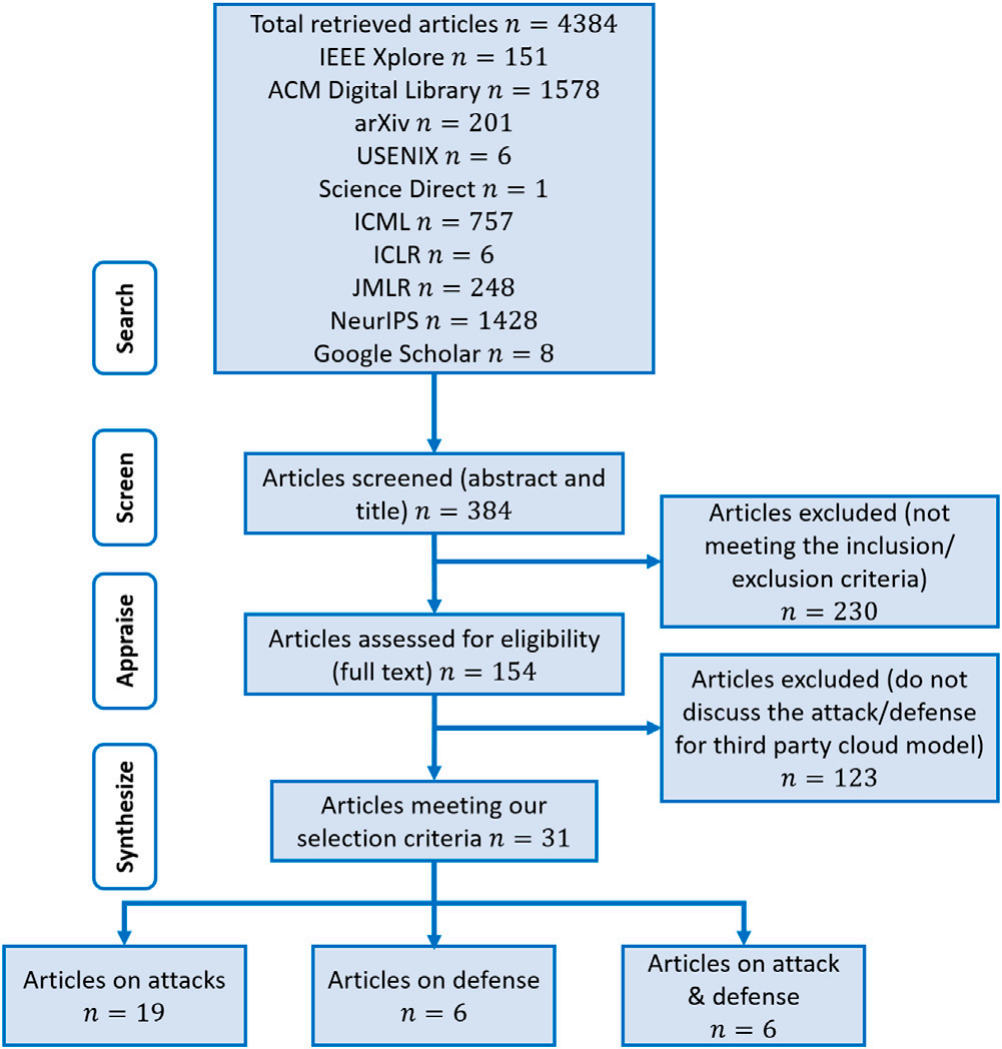
Search queries used to identify publications to include in the systematic review.

The search using the aforementioned strategy identified a total of 4,384 articles. After removing duplicate articles, title, and abstract screening, the overall number of articles reduced to 384. A total of 230 articles did not meet the inclusion criteria and were therefore excluded. From the remaining 154 articles, 123 articles did not discuss attack/defense for third-party cloud-hosted ML models and were excluded as well. Of the remaining articles, a total of 31 articles are identified as relevant. Reasons for excluding articles were documented and reported in a PRISMA flow diagram, depicted in Figure 4. These articles were categorized into three classes, that is, articles that are specifically focused on attacks, articles that are specifically focused on defenses, and articles that considered both attacks and defenses containing 19, 6, and 6 articles each, respectively.

### 3.2 Overview of the Selected Studies

**FIGURE 5:**
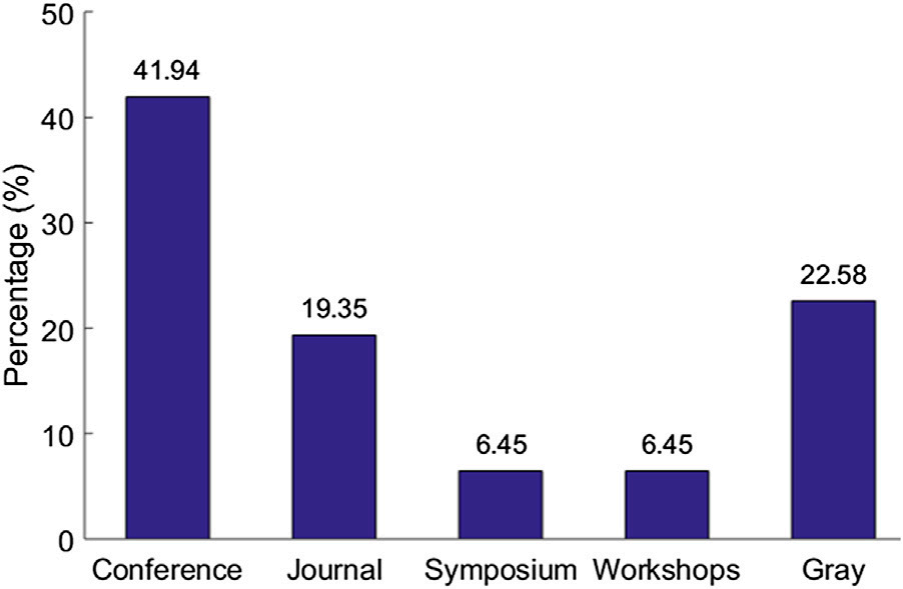
Flowchart of systematic review and categorization.

**FIGURE 6:**
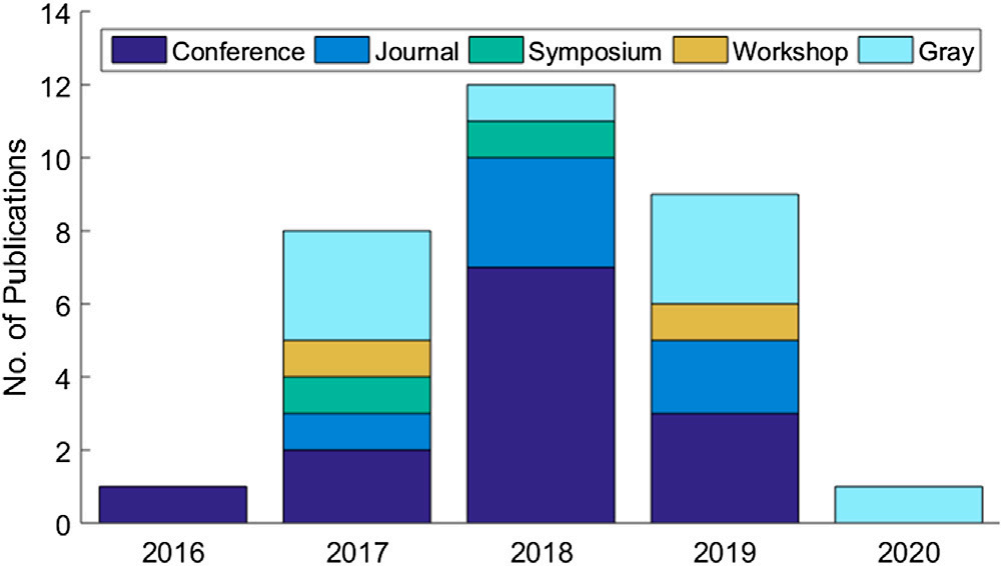
Distribution of selected publications according to their types.

**FIGURE 7:**
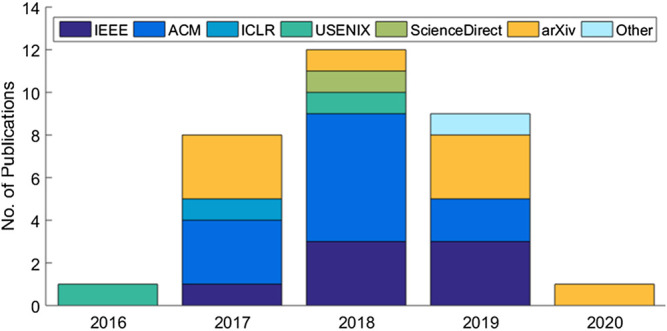
Distribution of selected publications by types over years.

The systematic review eventually identified a set of 31 articles related to cloud-based ML/DL models and MLaaS, which we categorized into three classes as mentioned above and shown in Figure 4. As shown in Figure 5, a significant portion of the selected articles were published in conferences (41.94%); comparatively, a very smaller proportion of these articles were published in journals or transactions (19.35%). The percentage of gray literature (i.e., non-peer–reviewed articles) is 25.81%. Yet, a very small proportion of publications are published in symposia (6.45%), and this percentage is the same for workshop papers. The distribution of selected publications by their types over the years is shown in Figure 6. The figure depicts that the interest in the security of cloud-hosted ML/DL models increased in the year 2017 and was at a peak in the year 2018 and was slightly lower in the year 2019 as compared to 2018. Also, the majority of the articles during these years were published in conferences. The distribution of selected publications by their publishers over the years is depicted in Figure 7, the figure shows that the majority of the publications have been published at IEEE, ACM, and arXiv. There is a similar trend in the number of articles in the year 2017, 2018, and 2019 as discussed previously.

### 3.3 Some Partially Related Non-Selected Studies: A Discussion

We have described our inclusion and exclusion criteria that help us to identify relevant articles. We note, however, that some seemingly relevant articles failed to meet the inclusion criteria. Here, we briefly describe few such articles for giving a rationale why they were not included.

- Liang et al. (2016) investigated the security challenges for the client-side classifiers via a case study on the Google’s phishing pages filter, a very widely used classifier for automatically detecting unknown phishing pages. They devised an attack that is not relevant to the cloud-based service.
- Demetrio et al. (2020) presented WAF-A-MoLE, a tool that models the presence of an adversary. This tool leverages a set of mutation operators that alter the syntax of a payload without affecting the original semantics. Using the results, the authors demonstrated that ML-based WAFs are exposed to a concrete risk of being bypassed. However, this attack is not associated with any cloud-based services.
- Authors in Apruzzese et al. (2019) discussed adversarial attacks where the machine learning model is compromised to induce an output favorable to the attacker. These attacks are realized in a different setting as compared to the scope of this systematic review, as we only included the articles which discuss the attack or defense when the cloud is outsourcing its services as MLaaS.
- Han et al. (2020) conducted the first systematic study of the practical traffic space evasion attack on learning-based network intrusion detection systems; again it is out of the inclusion criteria of our work.
- Chen et al. (2018) designed and evaluated three types of attackers targeting the training phases to poison our detection. To address this threat, the authors proposed the detection system, KuafuDet, and showed it significantly reduces false negatives and boosts the detection accuracy.
- Song et al. (2020) presented a federated defense approach for mitigating the effect of adversarial perturbations in a federated learning environment. This article can be potentially relevant for our study as they address the problem of defending cloud-hosted ML models; however, instead of using a third-party service, the authors conducted the experiments on a single computer system in a simulated environment; therefore, this study is not included in the analysis of this article.
- In a similar study, Zhang et al. (2019) presented a defense mechanism for defending adversarial attacks on cloud-aided automatic speech recognition (ASR); however, it is not explicitly stated that the cloud is outsourcing ML services and also which ML/DL model or MLaaS was used in experiments.

## 4 Attacks on Cloud-Hosted Machine Learning Models (Q1)

In this section, we present the findings from the systematically selected articles that aim at attacking cloud-hosted/third-party ML/DL models.

### 4.1 Attacks on Cloud-Hosted Machine Learning Models: Thematic Analysis

In ML practice, it is very common to outsource the training of ML/DL models to third-party services that provide high computational resources on the cloud. Such services enable ML practitioners to upload their models along with training data which is then trained on the cloud. Although such services have clear benefits for reducing the training and inference time; however, these services can easily be compromised and to this end, different types of attacks against these services have been proposed in the literature. In this section, we present the thematic analysis of 19 articles that are focused on attacking cloud-hosted ML/DL models. These articles are classified into five major themes: 1) attack type, 2) threat model, 3) attack method, 4) target model(s), and 5) dataset.

*Attack type:* A wide variety of attacks have been proposed in the literature. These are listed below with their descriptions provided in the next section.

- Adversarial attacks (Brendel et al., 2017);
- Backdoor attacks^6^ (Chen et al., 2017; Gu et al., 2019);
- Cyber kill chain–based attack (Nguyen, 2017);
- Data manipulation attacks (Liao et al., 2018);
- Evasion attacks (Hitaj et al., 2019);
- Exploration attacks (Sethi and Kantardzic, 2018);
- Model extraction attacks (Correia-Silva et al., 2018; Kesarwani et al., 2018; Joshi and Tammana, 2019; Reith et al., 2019);
- Model inversion attacks (Yang et al., 2019);
- Model-reuse attacks (Ji et al., 2018);
- Trojan attacks (Liu et al., 2018).

*Threat model:* Cloud ML attacks are based on different threat models, with the salient types with examples are listed below.

- black box attacks (no knowledge) (Brendel et al., 2017; Chen et al., 2017; Hosseini et al., 2017; Correia-Silva et al., 2018; Sethi and Kantardzic, 2018; Hitaj et al., 2019);
- white box attacks (full knowledge) (Liao et al., 2018; Liu et al., 2018; Gu et al., 2019; Reith et al., 2019);
- gray box attacks (partial knowledge) (Ji et al., 2018; Kesarwani et al., 2018).

*Attack method:* In each article, a different type of method is proposed for attacking cloud-hosted ML/DL models; a brief description of these methods is presented in Table 1 and is discussed in detail in the next section.

**TABLE 1:** Summary of the state-of-the art attack types for cloud-based/third-party ML/DL models.

Author(s)	Attack type	Method	Target model (s)	Threat model	Data
(Brendel et al., 2017)	Adversarial attack	Presented a decision-based attack, i.e., the boundary attack	Two ML classifiers from Clarifai.com, i.e., brand and celebrity recognition	Black box	Two datasets: Natural images and celebrities
(Saadatpanah et al., 2019)	—	Crafted adversarial examples for copyright detection system	YouTube content ID and AudioTag copyright	White box and black box	N/A
(Hosseini et al., 2017)	—	Proposed two targeted attacks for video labeling and shot detection	Google cloud video intelligence API	Black box	—
(Kesarwani et al., 2018)	Extraction attack	Used information gain to measure model learning rate	Decision tree deployed on BigML platform	Gray box	Four BigML datasets, IRS tax pattern, GSS survey, email importance, steak survey
(Correia-Silva et al., 2018)	—	Knowledge extraction by querying the model with unlabeled data samples and then used responses to create fake dataset and model	Three local CNN models for visual recognition for facial expression, object, and crosswalk classification and Microsoft Azure Emotion API	Black box	Used three datasets for facial expression recognition, object, and satellite crosswalk classification
(Reith et al., 2019)	—	Performed model extraction attacks on the homomorphic encryption-based protocol for preserving SVR-based indoor localization	Support vector regressor (SVR) and SVM	White box	California housing, Boston house prices, UJIIndoorLoc, and IPIN 2016 tutorial
(Joshi and Tammana, 2019)	—	Proposed a variant of gradient driven adaptive learning rate (GDALR) for stealing MLaaS models	Used three different models	Black box	Iris, liver disease, and land satellite datasets
(Sethi and Kantardzic, 2018)	Exploration attack	Presented a seed-explore-exploit framework for generating adversarial samples	Google cloud prediction platform	Black box	10 real-world datasets
(Gu et al., 2019)	Backdoor attack	Realized attack by poisoning training samples and labels	MNIST and a U.S. street sign classifier, i.e., Faster-RCNN with outsourced training and transfer learning	White box	MNIST and U.S. traffic signs dataset
(Chen et al., 2017)	—	Used poisoning strategies to realized a targeted attack and proposed two types of backdoor poisoning attacks	Two face recognition models, i.e., DeepID and VGG-Face	Black box	YouTube aligned face dataset
(Liu et al., 2018)	Trojan attack	Proposed stealth infection on neural network-based Trojan attack	Cloud-based intelligent supply chain, i.e., MLaaS	White box	Fashion-MNIST
(Gong et al., 2019)	—	Proposed real-time adversarial example crafting procedure	Voice/speech enabled devices and Google Speech	Gray box	Voice-command dataset
(Ji et al., 2018)	Model reuse attack	Presented empirical evaluation of model-reuse attacks on primitive models and realizing attack by generating semantically similar neighbors and identifying salient features	Pretrained primitive models for speech recognition, autonomous steering, face verification, and skin cancer screening	Gray box	Speech commands, udacity self-driving car challenge, VGG Face2, and International Skin Imaging Collaboration (ISIC) datasets
(Liao et al., 2018)	Data manipulation attack	Studied data manipulation attacks for stealthily manipulating ML and DL models using transfer learning and gradient descent	Cloud-hosted ML and DL models	White box	Enron spam and MINIST
(Sehwag et al., 2019)	—	Crafted out-of-distribution exploratory adversarial examples to compromise ML/DL models of Clarifai’s content moderation system in the cloud	Cloud-hosted ML and DL models	White box and black box	MINIST, CIFAR, and ImageNet
(Nguyen, 2017)	Cyber kill chain attack	Proposed a high-level threat model for ML cyber kill chain and provided proof of concept	IBM visual recognition MLaaS (i.e., cognitive classifier for classification cats and female lions)	N/A	Project Wolf Eye
(Hilprecht et al., 2019)	Membership inference attack	Monte Carlo based attack and membership inference attack on GAN.	Amazon web services p2	Black box	MNIST, fashion-MNIST, and CIFAR
(Hitaj et al., 2019)	Evasion attacks	Realized evasion attacks using two ensemble neural networks	Watermarking detection models	Black box	MNIST
(Yang et al., 2019)	Iversion attacks	Constructed an auxiliary set for training the inversion model	CNN	Gray-box	FaceScrub, CelebA, and CIFAR-10
	—	—	—	—	—

*Target model(s):* Considered studies have used different MLaaS services (e.g., Google Cloud ML Services (Hosseini et al., 2017; Salem et al., 2018; Sethi and Kantardzic, 2018), ML models of BigML Platform (Kesarwani et al., 2018), IBM’s visual recognition (Nguyen, 2017), and Amazon Prediction APIs (Reith et al., 2019; Yang et al., 2019)).

*Dataset:* These attacks have been realized using different datasets ranging from small size datasets (e.g., MNIST (Gu et al., 2019) and Fashion-MNIST (Liu et al., 2018)) to large size datasets (e.g., YouTube Aligned Face Dataset (Chen et al., 2017), Project Wolf Eye (Nguyen, 2017), and Iris dataset (Joshi and Tammana, 2019)). Other datasets include California Housing, Boston House Prices, UJIIndoorLoc, and IPIN 2016 Tutorial (Reith et al., 2019), FaceScrub, CelebA, and CIFAR-10 (Yang et al., 2019). A summary of thematic analyses of these attacks is presented in Table 1 and briefly described in the next section.

### 4.2 Taxonomy of Attacks on Cloud-Hosted Machine Learning Models

**FIGURE 8:**
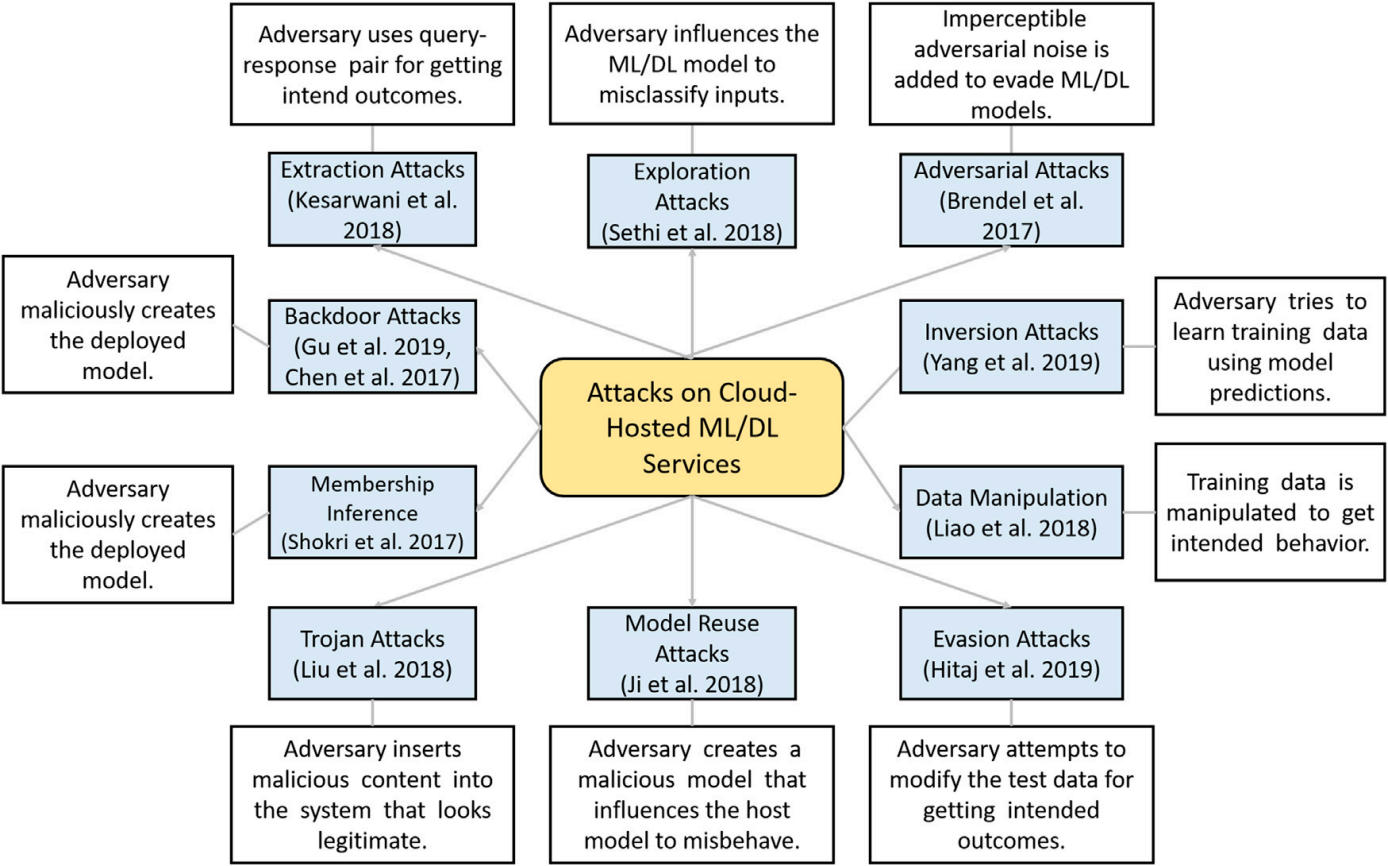
Distribution of selected publications by publishers over years.

In this section, we present a taxonomy and description of different attacks described above in thematic analysis. A taxonomy of attacks on cloud-hosted ML/DL models is depicted in Figure 8 and is described next.

#### 4.2.1 Adversarial Attacks

In recent years, DL models have been found vulnerable to carefully crafted imperceptible adversarial examples (Goodfellow et al., 2014). For instance, a decision-based adversarial attack namely *the boundary attack* against two black box ML models trained for brand and celebrity recognition hosted at Clarifai.com are proposed in (Brendel et al., 2017). The first model identifies brand names from natural images for 500 distinct brands and the second model recognizes over 10,000 celebrities. To date, a variety of adversarial examples generation methods have been proposed in the literature so far, the interesting readers are referred to recent surveys articles for detailed taxonomy of different types of adversarial attacks (i.e., Akhtar and Mian, 2018; Yuan et al., 2019; Qayyum et al., 2020b; Demetrio et al., 2020).

#### 4.2.2 Exploratory Attacks

These attacks are inference time attacks in which adversary attempts to evade the underlying ML/DL model, for example, by forcing the classifier (i.e., ML/DL model) to misclassify a positive sample as a negative one. Exploratory attacks do not harm the training data and only affects the model at test time. A data-driven exploratory attack using the *Seed*–*Explore*–*Exploit* strategy for evading Google’s cloud prediction API considering black box settings is presented in (Sethi and Kantardzic, 2018). The performance evaluation of the proposed framework was performed using 10 real-world datasets.

#### 4.2.3 Model Extraction Attacks

In model extraction attacks, adversaries can query the deployed ML model and can use query–response pair for compromising future predictions and also, they can potentially realize privacy breaches of the training data and can steal the model by learning extraction queries. In Kesarwani et al. (2018), the authors presented a novel method for quantifying the extraction status of models for users with an increasing number of queries, which aims to measure model learning rate using information gain observed by query and response streams of users. The key objective of the authors was to design a cloud-based system for monitoring model extraction status and warnings. The performance evaluation of the proposed method was performed using a decision tree model deployed on the BigML MLaaS platform for different adversarial attack scenarios. Similarly, a model extraction/stealing strategy is presented by Correia-Silva et al. (2018). The authors queried the cloud-hosted DL model with random unlabeled samples and used their predictions for creating a fake dataset. Then they used the fake dataset for building a fake model by training an oracle (copycat) model in an attempt to achieve similar performance as of the target model.

#### 4.2.4 Backdooring Attacks

In backdooring attacks, an adversary maliciously creates the trained model which performs as good as expected on the users’ training and validation data, but it performs badly on attacker input samples. The backdooring attacks on deep neural networks (DNNs) are explored and evaluated in (Gu et al., 2019). The authors first explored the properties of backdooring for a toy example and created a backdoor model for handwritten digit classifier and then demonstrated that backdoors are powerful for DNN by creating a backdoor model for a United States street sign classifier. Where, two scenarios were considered, that is, outsourced training of the model and transfer learning where an attacker can acquire a backdoor pretrained model online. In another similar study (Chen et al., 2017), a targeted backdoor attack for two state-of-the art face recognition models, that is, DeepID (Sun et al., 2014) and VGG-Face (Parkhi et al., 2015) is presented. The authors proposed two categories of backdooring poisoning attacks, that is, input–instance–key attacks and pattern–key attacks using two different data poising strategies, that is, input–instance–key strategies and pattern–key strategies, respectively.

#### 4.2.5 Trojan Attacks

In Trojan attacks, the attacker inserts malicious content into the system that looks legitimate but can take over the control of the system. However, the purpose of Trojan insertion can be varied, for example, stealing, disruption, misbehaving, or getting intended behavior. In Liu et al. (2018), the authors proposed a stealth infection on neural networks, namely, SIN2 to realize a practical supply chain triggered neural Trojan attacks. Also, they proposed a variety of Trojan insertion strategies for agile and practical Trojan attacks. The proof of the concept is demonstrated by developing a prototype of the proposed neural Trojan attack (i.e., SIN2) in Linux sandbox and used Torch (Collobert et al., 2011) ML/DL framework for building visual recognition models using the Fashion-MNIST dataset.

#### 4.2.6 Model-Reuse Attacks

In model-reuse attacks, an adversary creates a malicious model (i.e., adversarial model) that influences the host model to misbehave on targeted inputs (i.e., triggers) in extremely predictable fashion, that is, getting a sample classified into specific (intended class). For instance, experimental evaluation of model-reuse attacks for four pretrained primitive DL models (i.e., speech recognition, autonomous steering, face verification, and skin cancer screening) is evaluated by Ji et al. (2018).

#### 4.2.7 Data Manipulation Attacks

Those attacks in which training data are manipulated to get intended behavior by the ML/DL model are known as data manipulation attacks. Data manipulation attacks for stealthily manipulating traditional supervised ML techniques and logistic regression (LR) and CNN models are studied by Liao et al. (2018). In the attack strategy, the authors added a new constraint on fully connected layers of the models and used gradient descent for retraining them, and other layers were frozen (i.e., were made non-trainable).

#### 4.2.8 Cyber Kill Chain–Based Attacks

Kill chain is a term used to define steps for attacking a target usually used in the military. In cyber kill chain–based attacks, the cloud-hosted ML/DL models are attacked, for example, a high-level threat model targeting ML cyber kill chain is presented by Nguyen (2017). Also, the authors provided proof of concept by providing a case study using IBM visual recognition MLaaS (i.e., cognitive classifier for classification cats and female lions) and provided recommendations for ensuring secure and robust ML.

#### 4.2.9 Membership Inference Attacks

In a typical membership inference attack, for given input data and black box access to the ML model, an attacker attempts to figure out if the given input sample was the part of the training set or not. To realize a membership inference attack against a target model, a classification model is trained for distinguishing between the predictions of the target model against the inputs on which it was trained and that those on which it was not trained (Shokri et al., 2017).

#### 4.2.10 Evasion Attacks

Evasion attacks are inference time attacks in which an adversary attempts to modify the test data for getting the intended outcome from the ML/DL model. Two evasion attacks against watermarking techniques for DL models hosted as MLaaS have been presented by Hitaj et al. (2019). The authors used five publicly available models and trained them for distinguishing between watermarked and clean (non-watermarked) images, that is, binary image classification tasks.

#### 4.2.11 Model Inversion Attacks

In model inversion attacks, an attacker tries to learn about training data using the model’s outcomes. Two model inversion techniques have been proposed by Yang et al. (2019), that is, training an inversion model using auxiliary set composed by utilizing adversary’s background knowledge and truncation-based method for aligning the inversion model. The authors evaluated their proposed methods on a commercial prediction MLaaS named Amazon Rekognition.

## 5 Toward Securing Cloud-Hosted Machine Learning Models (Q2)

In this section, we present the insights from the systematically selected articles that provide tailored defense against specific attacks and report the articles that along with creating attacks propose countermeasure for the attacks for cloud-hosted/third-party ML/DL models.

### 5.1 Defenses for Attacks on Cloud-Hosted Machine Learning Models: Thematic Analysis

Leveraging cloud-based ML services for computational offloading and minimizing the communication overhead is accepted as a promising trend. While cloud-based prediction services have significant benefits, however, by sharing the model and the training data raises many privacy and security challenges. Several attacks that can compromise the model and data integrity, as described in the previous section. To avoid such issues, users can download the model and make inferences locally. However, this approach has certain drawbacks, including, confidentiality issues, service providers cannot update the models, adversaries can use the model to develop evading strategies, and privacy of the user data is compromised. To outline the countermeasures against these attacks, we present the thematic analysis of six articles that are focused on defense against the tailored attacks for cloud-hosted ML/DL models or data. In addition, we also provide the thematic analysis of those six articles that propose defense against specific attacks. These articles are classified into five major themes: 1) attack type, 2) defense, 3) target model(s), 4) dataset, and 5) measured outcomes. The thematic analysis of these systematically reviewed articles that are focused on developing defense strategies against attacks is given below.

*Considered attacks for developing defenses:* The defenses proposed in the reviewed articles are developed against the following specific attacks.

- Extraction attacks (Tramèr et al., 2016; Liu et al., 2017);
- Inversion attacks (Liu et al., 2017; Sharma and Chen, 2018);
- Adversarial attacks (Hosseini et al., 2017; Wang et al., 2018b; Rouhani et al., 2018);
- Evasion attacks (Lei et al., 2020);
- GAN attacks (Sharma and Chen, 2018);
- Privacy threat attacks (Hesamifard et al., 2017);
- ide channel and cache-timing attacks (Jiang et al., 2018);
- Membership inference attacks (Shokri et al., 2017; Salem et al., 2018).

Most of the aforementioned attacks are elaborated in previous sections. However, in the selected articles that are identified as either defense or attack and defense articles, some attacks are specifically created, for instance, GAN attacks, side channel, cache-timing attack, privacy threats, etc. Therefore, the attacks are worth mentioning in this section to explain the specific countermeasures proposed against them in the defense articles.

*Defenses against different attacks:* To provide resilience against these attacks, the authors of selected articles proposed different defense algorithms, which are listed below against each type of attack.

- Extraction attacks: MiniONN (Liu et al., 2017), rounding confidence, differential, and ensemble methods (Tramèr et al., 2016);
- Adversarial attacks: ReDCrypt (Rouhani et al., 2018) and Arden (Wang et al., 2018b);
- Inversion attacks: MiniONN (Liu et al., 2017) and image disguising techniques (Sharma and Chen, 2018);
- Privacy attacks: encryption-based defense (Hesamifard et al., 2017; Jiang et al., 2018);
- Side channel and cache-timing attacks: encryption-based defense (Hesamifard et al., 2017; Jiang et al., 2018);
- Membership inference attack: dropout and model stacking (Salem et al., 2018).

*Target model(s):* Different cloud-hosted ML/DL models have been used for the evaluation of the proposed defenses, as shown in Table 2.

**TABLE 2:** Summary of attack types and corresponding defenses for cloud-based/third-party ML/DL models.

Author	Attack	Defense	Target model	Data	Measured outcomes
(Liu et al., 2017)	Extraction attack and inversion attack	MiniONN: a defense against information leakage in DNN to transform into an oblivious NN	Cloud-hosted DL models, neural network for cloud-based prediction services	MNIST and CIFAR-10	Response latency and message sizes
(Rouhani et al., 2018)	Adversarial attacks	ReDCrypt: reconfigurable hardware-accelerated framework for the privacy-preserving	Cloud-hosted DL models	MNIST and MovieLens	Throughput
(Wang et al., 2018b)	—	Arden: To distribute DNN model computation among edge device and cloud data centers	Partial cloud-hosted DNN models	MNIST, SVHN, and CIFAR-10	Latency, accuracy, and privacy budget
(Hosseini et al., 2017)	—	Incorporating randomness to video analysis algorithms	Google cloud video intelligence API	Videos comprising of adversarial examples	Histogram peaks to detect shot change
(Sharma and Chen, 2018)	Inversion attack and GAN attack	Image disguising techniques to ensure the protection against model-based adversarial attacks	Cloud-hosted DL models	MNIST and CIFAR-10	Accuracy, average visual privacy, and Fano factor
(Hesamifard et al., 2017)	Privacy threats due to raw cloud data	Homomorphic encryption to preserve the privacy and integrity of data in DNN	Cloud-based DNN	Crab dataset, fertility dataset, climate dataset	Accuracy and training time
(Jiang et al., 2018)	Side channel and cache-timing attack	Secure logistic encryption along with hardware-based security enhancement by exploiting software guard extensions	Cloud-hosted LR models	Edinburgh MI, WI-Breast cancer, and MONK’s prob	Area under the curve, complexity, and model training time
(Lei et al., 2020)	Evasion attack	Pelican: similarity-based analysis of unknown website with the known phishing Web site	BitDefender’s partical processing hosted on cloud	PhishTank, PhishNet	Similarity index
(Tramèr et al., 2016)	Extraction attack	Rounding confidences to some precision, differential privacy to protect training data elements, ensemble methods	ML models hosted on BigML and amazon	102 categories flower dataset, face dataset, iris dataset, and traffic signs dataset	Success rate given the perturbation budget
(Shokri et al., 2017)	Membership inference attack	Top *k* class model predictions, increase entropy, regularization and reducing precision of prediction vector	MLaaS classification models of Google and Amazon APIs	CIFAR-10,purchases, locations, Texas hospital stays, MNIST, UCI adults	Accuracy and precision
(Salem et al., 2018)	—	Dropout and model stacking to prevent overfitting	Google cloud prediction API	Used eight different datasets	Precision and recall
(Wang et al., 2018a)	Misclassification attacks	Neuron distance model, ensemble method, dropout randomization	Google cloud ML, microsoft cognitive toolkit (CNTK), and the PyTorch	102-Class VGG flower, face dataset, iris dataset, and traffic signs dataset, Google’s InceptionV3	Accuracy and success rate

*Dataset(s) used:* The robustness of these defenses have been evaluated using various datasets ranging from small size datasets (e.g., MNIST (Liu et al., 2017; Wang et al., 2018b; Rouhani et al., 2018; Sharma and Chen, 2018)) and CIFAR-10 (Liu et al., 2017; Wang et al., 2018b; Sharma and Chen, 2018)), to large size datasets (e.g., Iris dataset (Tramèr et al., 2016), fertility and climate dataset (Hesamifard et al., 2017), and breast cancer (Jiang et al., 2018)). Other datasets include Crab dataset (Hesamifard et al., 2017), Face dataset, Traffic signs dataset, Traffic signs dataset (Tramèr et al., 2016), SVHN (Wang et al., 2018b), Edinburgh MI, Edinburgh MI, WI-Breast Cancerband MONKs Prob (Jiang et al., 2018), crab dataset, fertility dataset, and climate dataset (Hesamifard et al., 2017). Each of the defense techniques discussed above is mapped in Table 2 to the specific attack for which it was developed.

*Measured outcomes:* The measured outcomes based on which the defenses are evaluated are response latency and message sizes (Liu et al., 2017; Wang et al., 2018b), throughput comparison (Rouhani et al., 2018), average on the cache miss rates per second (Sharma and Chen, 2018), AUC, space complexity to demonstrate approximated storage costs (Jiang et al., 2018), classification accuracy of the model as well as running time (Hesamifard et al., 2017; Sharma and Chen, 2018), similarity index (Lei et al., 2020), and training time (Hesamifard et al., 2017; Jiang et al., 2018).

### 5.2 Taxonomy of Defenses on Cloud-Hosted Machine Learning Model Attacks

**FIGURE 9:**
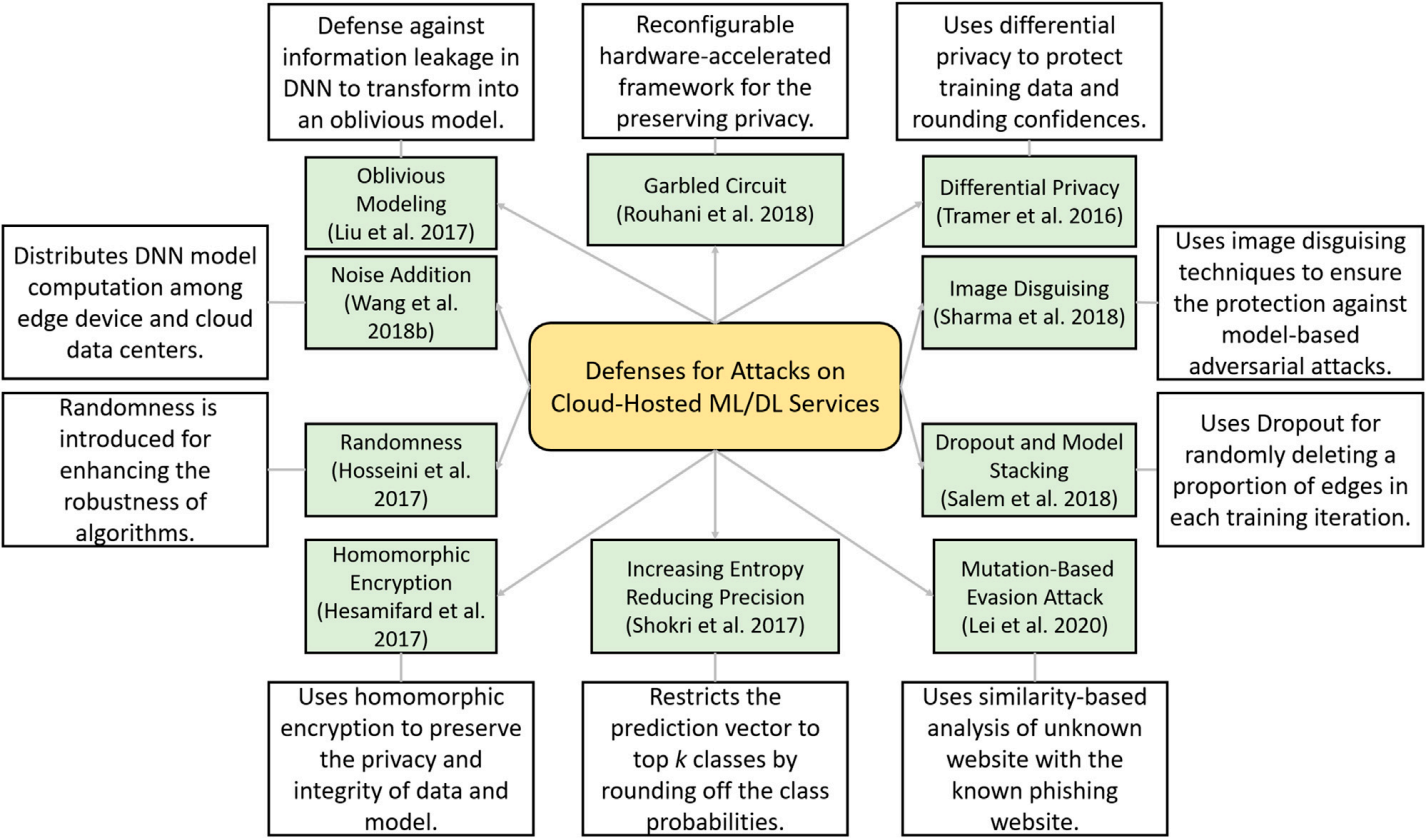
Taxonomy of different attacks realized on the third-party cloud-hosted machine learning (ML) or deep learning (DL) models.

In this section, we present a taxonomy and summary of different defensive strategies against attacks on cloud-hosted ML/DL models as described above in thematic analysis. A taxonomy of these defenses strategies is presented in Figure 9 and is described next.

#### 5.2.1 MiniONN

DNNs are vulnerable to model inversion and extraction attacks. Liu et al. (2017) proposed that without making any changes to the training phase of the model it is possible to change the model into an oblivious neural network. They make the nonlinear function such as *tanh* and *sigmoid* function more flexible, and by training the models on several datasets, the authors demonstrated significant results with minimal loss in the accuracy. In addition, they also implemented the offline precomputation phase to perform encryption incremental operations along with the SIMD batch processing technique.

#### 5.2.2 ReDCrypt

A reconfigurable hardware-accelerated framework is proposed by Rouhani et al. (2018), for protecting the privacy of deep neural models in cloud networks. The authors perform an innovative and power-efficient implementation of Yao’s Garbled Circuit (GC) protocol on FPGAs for preserving privacy. The proposed framework is evaluated for different DL applications, and it has achieved up to 57-fold throughput gain per core.

#### 5.2.3 Arden

To offload the large portion of DNNs from the mobile devices to the clouds and to make the framework secure, a privacy-preserving mechanism Arden is proposed by Wang et al. (2018b). While uploading the data to the mobile-cloud perturbation, noisy samples are included to make the data secure. To verify the robustness, the authors perform rigorous analysis based on three image datasets and demonstrated that this defense is capable to preserve the user privacy along with inference performance.

#### 5.2.4 Image Disguising Techniques

While leveraging services from the cloud GPU server, the adversary can realize an attack by introducing malicious created training data, perform model inversion, and use the model for getting desirable incentives and outcomes. To protect from such attacks and to preserve the data as well as the model, Sharma and Chen (2018) proposed an image disguising mechanism. They developed a toolkit that can be leveraged to calibrate certain parameter settings. They claim that the disguised images with block-wise permutation and transformations are resilient to GAN-based attack and model inversion attacks.

#### 5.2.5 Homomorphic Encryption

For making the cloud services of outsourced MLaaS secure, Hesamifard et al. (2017) proposed a privacy-preserving framework using homomorphic encryption. They trained the neural network using the encrypted data and then performed the encrypted predictions. The authors demonstrated that by carefully choosing the polynomials of the activation functions to adopt neural networks, it is possible to achieve the desired accuracy along with privacy-preserving training and classification.

In a similar study, to preserve the privacy of outsourced biomedical data and computation on public cloud servers, Jiang et al. (2018) built a homomorphically encrypted model that reinforces the hardware security through Software Guard Extensions. They combined homomorphic encryption and Software Guard Extensions to devise a hybrid model for the security of the most commonly used model for biomedical applications, that is, LR. The robustness of the Secure LR framework is evaluated on various datasets, and the authors also compared its performance with state-of-the-art secure LR solutions and demonstrated its superior efficiency.

#### 5.2.6 Pelican

Lei et al. (2020) proposed three mutation-based evasion attacks and a sample-based collision attack in white-, gray-, and black box scenarios. They evaluated the attacks and demonstrated a 100% success rate of attack on Google’s phishing page filter classifier, while a success rate of up to 81% for the transferability on Bitdefender TrafficLight. To deal with such attacks and to increase the robustness of classifiers, they proposed a defense method known as Pelican.

#### 5.2.7 Rounding Confidences and Differential Privacy

Tramèr et al. (2016) presented the model extraction attacks against the online services of BigML and Amazon ML. The attacks are capable of model evasion, monetization, and can compromise the privacy of training data. The authors also proposed and evaluated countermeasures such as rounding confidences against equation-solving and decision tree pathfinding attacks; however, this defense has no impact on the regression tree model attack. For the preservation of training data, differential privacy is proposed; this defense reduces the ability of an attacker to learn insights about the training dataset. The impact of both defenses is evaluated on the attacks for different models, while the authors also proposed ensemble models to mitigate the impact of attacks; however, their resilience is not evaluated.

#### 5.2.8 Increasing Entropy and Reducing Precision

The training of attack using shadow training techniques against black box models in the cloud-based Google Prediction API and Amazon ML models are studied by Shokri et al. (2017). The attack does not require prior knowledge of training data distribution. The authors emphasize that in order to protect the privacy of medical-related datasets or other public-related data, countermeasures should be designed. For instance, restriction of prediction vector to top *k* classes, which will prevent the leakage of important information or rounding down or up the classification probabilities in the prediction. They show that regularization can be effective to cope with overfitting and increasing the randomness of the prediction vector.

#### 5.2.9 Dropout and Model Stacking

In the study by Salem et al. (2018), the authors created three diverse attacks and tested the applicability of these attacks on eight datasets from which six are similar as used by Shokri et al. (2017), whereas in this work, news dataset and face dataset is included. In the threat model, the authors considered black box access to the target model which is a supervised ML classifier with binary classes that was trained for binary classification. To mitigate the privacy threats, the authors proposed a dropout-based method which reduces the impact of an attack by randomly deleting a proportion of edges in each training iteration in a fully connected neural network. The second defense strategy is model stacking, which hierarchically organizes multiple ML models to avoid overfitting. After extensive evaluation, these defense techniques showed the potential to mitigate the performance of the membership inference attack.

#### 5.2.10 Randomness to Video Analysis Algorithms

Hosseini et al. designed two attacks specifically to analyze the robustness of video classification and shot detection (Hosseini et al., 2017). The attack can subtly manipulate the content of the video in such a way that it is undetected by humans, while the output from the automatic video analysis method is altered. Depending on the fact that the video and shot labels are generated by API by processing only the first video frame of every second, the attack can successfully deceive API. To deal with the shot removal and generation attacks, the authors proposed the inclusion of randomness for enhancing the robustness of algorithms. However, in this article, the authors thoroughly evaluated the applicability of these attacks in different video setting, but the purposed defense is not rigorously evaluated.

#### 5.2.11 Neuron Distance Threshold and Obfuscation

Transfer learning is an effective technique for quickly building DL student models in which knowledge from a Teacher model is transferred to a Student model. However, Wang et al. (2018a) discussed that due to the centralization of model training, the vulnerability against misclassification attacks for image recognition on black box Student models increases. The authors proposed several defenses to mitigate the impact of such an attack, such as changing the internal representation of the Student model from the Teacher model. Other defense methods include increasing dropout randomization which alters the student model training process, modification in input data before classification, adding redundancy, and using orthogonal model against transfer learning attack. The authors analyzed the robustness of these attacks and demonstrated that the neuron distance threshold is the most effective in obfuscating the identity of the Teacher model.

## 6 Pitfalls and Limitations

### 6.1 Lack of Attack Diversity

The attacks presented in the selected articles have limited scope and lack diversity, that is, they are limited to a specific setting, and the variability of attacks is limited as well. However, the diversity of attacks is an important consideration for developing robust attacks from the perspective of adversaries, and it ensures the detection and prevention of the attacks to be difficult. The diversity of attacks ultimately helps in the development of robust defense strategies. Moreover, the empirical evaluation of attack variabilities can identify the potential vulnerabilities of cybersecurity systems. Therefore, to make a more robust defense solution, it is important to test the model robustness under a diverse set of attacks.

### 6.2 Lack of Consideration for Adaptable Adversaries

Most of the defenses in the systematically reviewed articles are proposed for a specific attack and did not consider the adaptable adversaries. On the other hand, in practice, the adversarial attacks are an arms race between attackers and defenders. That is, the attackers continuously evolve and enhance their knowledge and attacking strategies to evade the underlying defensive system. Therefore, the consideration of adaptable adversaries is crucial for developing a robust and long-lasting defense mechanism. If we do not consider this, the adversary will adapt to our defensive system over time and will bypass it to get the intended behavior or outcomes.

### 6.3 Limited Progress in Developing Defenses

From the systematically selected articles that are collected from different databases, only 12 articles have presented defense methods for the proposed attack as compared to the articles that are focused on attacks, that is, 19. In these 12 articles, six have only discussed/presented a defense strategy and six have developed a defense against a particular attack. This indicates that there is limited activity from the research community in developing defense strategies for already proposed attacks in the literature. In addition, the proposed defenses only mitigate or detect those attacks for which they have been developed, and therefore, they are not generalizable. On the contrary, the increasing interest in developing different attacks and the popularity of cloud-hosted/third-party services demand a proportionate amount of interest in developing defense systems as well.

## 7 Open Research Issues

### 7.1 Adversarially Robust Machine Learning Models

In recent years, adversarial ML attacks have emerged as a major panacea for ML/DL models and the systematically selected articles have highlighted the threat of these attacks for cloud-hosted Ml/DL models as well. Moreover, the diversity of these attacks is drastically increasing as compared with the defensive strategies that can pose serious challenges and consequences for the security of cloud-hosted ML/DL models. Each defense method presented in the literature so far has been shown resilient to a particular attack which is realized in specific, settings and it fails to withstand for yet stronger and unseen attacks. Therefore, the development of adversarially robust ML/DL models remains an open research problem, while the literature suggests that worst-case robustness analysis should be performed while considering adversarial ML settings (Qayyum et al., 2020a; Qayyum et al., 2020b; Ilahi et al., 2020). In addition, it has been argued in the literature that most of ML developers and security incident responders are unequipped with the required tools for securing industry-grade ML systems against adversarial ML attacks Kumar et al. (2020). This indicates the increasing need for the development of defense strategies for securing ML/DL models against adversarial ML attacks.

### 7.2 Privacy-Preserving Machine Learning Models

In cloud-hosted ML services, preserving user privacy is fundamentally important and is a matter of high concern. Also, it is desirable that ML models built using users’ data should not learn information that can compromise the privacy of the individuals. However, the literature on developing privacy-preserving ML/DL models or MLaaS is limited. On the other hand, one of the privacy-preserving techniques that have been used for privacy protection for building a defense system for cloud-hosted ML/DL models, that is, the homomorphic encryption-based protocol (Jiang et al., 2018), has been shown vulnerable to model extraction attack (Reith et al., 2019). Therefore, the development of privacy-preserving ML models for cloud computing platforms is another open research problem.

### 7.3 Proxy Metrics for Evaluating Security and Robustness

From systematically reviewed literature on the security of cloud-hosted ML/DL models, we orchestrate that the interest from the research community in the development of novel security-centric proxy metrics for the evaluation of security threats and model robustness of cloud-hosted models is very limited. However, with the increasing proliferation of cloud-hosted ML services (i.e., MLaaS) and with the development/advancements of different attacks (e.g., adversarial ML attacks), the development of effective and scalable metrics for evaluating the robustness ML/DL models toward different attacks and defense strategies is required.

## 8 Threats to Validity

We now briefly reflect on our methodology in order to identify any threats to the validity of our findings. First, internal validity is maintained as the research questions we pose in Section 2.2 capture the objectives of the study. Construct validity relies on a sound understanding of the literature and how it represents the state of the field. A detailed study of the reviewed articles along with deep discussions between the members of the research team helped ensure the quality of this understanding. Note that the research team is of diverse skills and expertise in ML, DL, cloud computing, ML/DL security, and analytics. Also, the inclusion and exclusion criteria (Section 2.3) help define the remit of our survey. Data extraction is prone to human error as is always the case. This was mitigated by having different members of the research team review each reviewed article. However, we did not attempt to evaluate the quality of the reviewed studies or validate their content due to time constraints. In order to minimize selection bias, we cast a wide net in order to capture articles from different communities publishing in the area of MLaaS via a comprehensive set of bibliographical databases without discriminating based on the venue/source.

## 9 Conclusion

In this article, we presented a systematic review of literature that is focused on the security of cloud-hosted ML/DL models, also named as MLaaS. The relevant articles were collected from eight major publishers that include ACM Digital Library, IEEE Xplore, ScienceDirect, international conference on machine learning, international conference on learning representations, journal of machine learning research, USENIX, neural information processing systems, and arXiv. For the selection of articles, we developed a review protocol that includes inclusion and exclusion formulas and analyzed the selected articles that fulfill these criteria across two dimensions (i.e., attacks and defenses) on MLaaS and provide a thematic analysis of these articles across five attack and five defense themes, respectively. We also identified the limitations and pitfalls from the reviewed literature, and finally, we have highlighted various open research issues that require further investigation.

## Data Availability Statement

The original contributions presented in the study are included in the article/supplementary material, further inquiries can be directed to the corresponding author/s.

## Author Contributions

AQ led the work in writing the manuscript and performed the annotation of the data and analysis as well. AI performed data acquisition, annotation, and analysis from four venues, and contributed to the paper write-up. MU contributed to writing a few sections, did annotations of papers, and helped in analysis. WI performed data scrapping, annotation, and analysis from four venues, and helped in developing graphics. All the first four authors validated the data, analysis, and contributed to the interpretation of the results. AQ and AI helped in developing and refining the methodology for this systematic review. JQ conceived the idea and supervises the overall work. JQ, YEK, and AF provided critical feedback and helped shape the research, analysis, and manuscript. All authors contributed to the final version of the manuscript.

## Conflict of Interest

The authors declare that the research was conducted in the absence of any commercial or financial relationships that could be construed as a potential conflict of interest.

## Footnotes

1 We use MLaaS to cover both ML and DL as a Service cloud provisions.

2
https://cloud.google.com/ml-engine/.

3 A popular Python library for DL.

4
https://azure.microsoft.com/en-us/services/machine-learning-service/.

5
https://docs.aws.amazon.com/dlami/latest/devguide/AML2_0.html.

6 Backdoor attacks on cloud-hosted models can be further categorized into three categories (Chen et al., 2020): 1) complete model–based attacks, 2) partial model–based attacks, and 3) model-free attacks).
